# Who Is Metabolizing What? Discovering Novel Biomolecules in the Microbiome and the Organisms Who Make Them

**DOI:** 10.3389/fcimb.2020.00388

**Published:** 2020-07-31

**Authors:** Sneha P. Couvillion, Neha Agrawal, Sean M. Colby, Kristoffer R. Brandvold, Thomas O. Metz

**Affiliations:** ^1^Biological Sciences Division, Pacific Northwest National Laboratory, Richland, WA, United States; ^2^Elson S. Floyd College of Medicine, Washington State University, Spokane, WA, United States

**Keywords:** microbiome, function, standards-free metabolomics, ABPP, human health

## Abstract

Even as the field of microbiome research has made huge strides in mapping microbial community composition in a variety of environments and organisms, explaining the phenotypic influences on the host by microbial taxa—both known and unknown—and their specific functions still remain major challenges. A pressing need is the ability to assign specific functions in terms of enzymes and small molecules to specific taxa or groups of taxa in the community. This knowledge will be crucial for advancing personalized therapies based on the targeted modulation of microbes or metabolites that have predictable outcomes to benefit the human host. This perspective article advocates for the combined use of standards-free metabolomics and activity-based protein profiling strategies to address this gap in functional knowledge in microbiome research via the identification of novel biomolecules and the attribution of their production to specific microbial taxa.

## Who is Doing What? The Conundrum of Linking Taxonomy and Function

A number of studies have shown that the microbiome composition in an individual's gut and other body sites is inherently dynamic and changes over time due to many factors, such as dietary changes, medical interventions (e.g., antibiotic use), other environmental exposures, childhood maturation, normal aging, and illness. A widespread approach in microbiome research has been to associate different diseases with alterations in the microbiome (dysbiosis), but directionality is often unknown and it is not clear if these changes are causal or simply associative (Olesen and Alm, 2016). Fluctuations in community composition do not necessarily indicate changes in community function or metabolic activity (Whidbey et al., 2019). In order to be able to design microbiome-modulation based therapies to improve human health, a deeper functional knowledge is required and comprising of (A) complete biochemical characterization of microbiome metabolites, (B) the proteins involved in their production, conversion or transport, (C) the microbial populations responsible for producing, utilizing or otherwise interacting with these molecules, and (D) their effect on host physiology.

The Human Microbiome Project characterized the microbial communities present in multiple body habitats in a large cohort of healthy subjects with both 16S and shotgun metagenomic data. Although extensive variability was observed in the taxonomic diversity, metabolic pathways were evenly encoded across both individual and body habitats, revealing functional plasticity in these ecosystems (Human Microbiome Project Consortium, 2012). The variation between individuals can arise from a large number of co-varying factors (e.g., host lifestyle, diet, cultural habits, host genetics, age, disease states, maternal transmission, family members, local environment etc.), (Schmidt et al., 2018). Population based analyses have shown that known factors that correlate with shifts in microbiome composition and structure collectively explain only a fraction of the interindividual variance (Falony et al., 2016; Zhernakova et al., 2016), underscoring the complexity of molecular mechanisms that likely govern host-microbiome interactions. The chemical space spanning these interactions is massive, dynamically changing and shaped by multiple, often confounding, factors. Humans are continually exposed to a large number of substances that are foreign to the body. Human milk is an important first “exposure” for breast-fed infants, rich in biologically active components (oligosaccharides, hormones, lipids etc.) and harboring its own microbiome. The milk microbiome has recently attracted much scientific attention, given its role in early establishment of the infant gut microbiome and maternal health (McGuire and McGuire, 2017; Ramani et al., 2018; Moossavi et al., 2019). Other exposures include a variety of chemical compounds present in the foods we eat and xenobiotics such as pharmaceutical drugs, cosmetics, and environmental pollutants. Some of these molecules that may not be bioactive in their original form make their way through the digestive tract and are bio-transformed by the gut microbiota into products that are biologically active and may have a beneficial or detrimental effect to the host. Enterohepatic circulation of drugs, bile acids, and other chemicals through biliary excretion, gut microbial biotransformation, and intestinal reabsorption can result in altered pharmacology and toxicology (Klaassen and Cui, 2015; Winston and Theriot, 2020). Many commonly prescribed drugs are known to be metabolically altered by the microbiome, significantly impacting their biological activity. Thus, the interindividual variability in gut microbial composition means that a drug's efficacy or toxicity can vary depending on an individual's unique microbiome.

## Direct Characterization of Taxon-Specific Function

Microorganisms interact with each other and the host physiology via small molecule metabolites. These include exogeneous small molecules, metabolites produced by the host, microbial biotransformation products and molecules synthesized *de novo* by the microbes. Metabolomics is a powerful tool for characterizing the diverse array of small molecule metabolites that take part in the complex interplay between the microbiota, host, and environment.

In addition to direct characterization of microbial metabolites, which are the downstream products of metabolism, it is imperative to link these metabolites back to enzymes and other functional proteins expressed by the microbiota and that interact with these molecules in some fashion. Although many core functions can be performed by a number of different microbial members of the community (functional redundancy), other specialized functions have been attributed to specific taxa. For example, the Cgr2 protein from a single species in the gut, *Eggerthella lenta* has been found to inactivate digoxin, a plant toxin, and a cardiac drug (Koppel et al., 2018). Speculations about the genesis of microbial metabolites can be made through employing conventional omics approaches such as metagenomics, metatranscriptomics, global metaproteomics, and metabolomics, to find correlative relationships, but abundance measurements do not establish a direct functional connection. Distinguishing active populations within the microbiome is important from a metabolic perspective because there may be microbial candidates that have all the prerequisites for a given activity, but conventional methods cannot determine if the system is functionally competent. The value of function based approaches is illustrated in the case of *E. lenta* where the mere presence of the microbe in the gut was not found to correlate with levels of drug inactivation (Saha et al., 1983; Haiser et al., 2013). Chemoproteomic tools that require activity for a protein to appear in the final readout can be used to investigate the functionally active proteome.

Next generation standards-free metabolomics can provide comprehensive coverage of the metabolome from a variety of sample types including feces, blood plasma, milk etc. Candidate metabolite features that are capable of indirectly or directly modulating the host phenotype can be selected from the metabolome using a variety of strategies that include statistical significance using comparative study design, pathway, and systems analysis (Guijas et al., 2018). Standards-free metabolomics can enable comprehensive, putative compound identification, greatly accelerating the selection of metabolites implicated in a disease state for example, which have shown strong correlation to dysbiosis in the microbiome, indicating their possible involvement in causing the host phenotype. These molecules are then targets for activity-based probe design for use in profiling aliquots of the same or similar samples to identify the microbes that make the enzymes or transporters that act on the molecules of interest. One can envision that a similar workflow could be used to understand the effect of a drug or a dietary compound of interest on the microbiome and host, where activity-based probes can be custom-designed for the drug and standards-free metabolomics along with molecular networking strategies can be used to profile downstream products of microbial and host metabolism of the compound. In this perspective review, we provide a detailed vision for the integration of metabolomics and activity-based protein profiling for identifying novel molecules in microbiomes and the organisms responsible for their synthesis or metabolism.

## Standards-Free Metabolomics and Computational Library Building

An in-depth understanding of the human microbiome's effect on host physiology at the biomolecular level will require tools to predict and measure molecules metabolized by the microbes. Untargeted mass spectrometry-based metabolomics measurements enable thousands of metabolite signals to be measured from a sample, which is helpful when investigating a very diverse and largely uncharacterized chemical space like the human gut. Comprehensive compound identification is a significant, long-standing bottleneck faced by the metabolomics community. Accurate identification of small molecule structures will be fundamental to understanding the role of various metabolites in modulating biological processes in the microbes and the host. In recent years, there has been growing interest in integrating ion mobility spectrometry (IMS) into current MS-based analytical methods (Lanucara et al., 2014; May and McLean, 2015; Paglia et al., 2015; Metz et al., 2017; Dodds and Baker, 2019). Using a multidimensional analytical platform such as LC-IMS-MS/MS, which combines IMS and tandem mass spectrometry, not only provides improved separation and dynamic range of detection but also gives the user an additional dimension of structural information for high confidence identifications. IMS is capable of separating stereoisomers and isobaric compounds and measures the physical-chemical property of collision cross section (CCS), which has been shown to be highly reproducible (Stow et al., 2017). For features detected in the human microbiome, for example, defined by relative retention time, CCS, *m/z*, and mass fragmentation patterns in LC-IMS-MS/MS, a putative identification can be made, or a candidate list narrowed, using reference values of known molecules (Paglia and Astarita, 2017; King et al., 2019; Nuñez et al., 2019). Armed with multiple pieces of experimental information on an unknown molecule, the next step is to query in-house reference libraries. Traditionally, these have been determined experimentally through analyses of authentic reference materials: purified and concentrated compounds of interest are analyzed for relevant chemical properties (Castle et al., 2006; Sumner et al., 2007). The number of standards that can be analyzed by any single laboratory is inherently limited due to a variety of reasons including cost, availability of authentic reference materials, and instrument time. As a work-around, commercial reference libraries and freely available online spectral databases exist to aid researchers in metabolite identification. However, even as databases with reference spectra continue to grow, the metabolome coverage represented is still only a fraction of all the possible molecules that can be detected in biological and environmental samples. Building these reference libraries experimentally is slow and expensive, particularly when considering chemical space has been estimated to contain up to 10^60^ unique molecules (Dobson, 2004). Community wide sharing and curation of metabolomics data and associated metadata, reference databases, computational tool development, and knowledge dissemination will continue to be crucial for accelerating metabolomics research (Wang et al., 2016, 2020; Picache et al., 2020) but the challenge of identifying unknown molecules, especially those for which reference standards do not exist, remains a major roadblock. As a result, there has been growing interest in what has been termed “standards free” approaches, wherein reference values are determined through *in silico* methods, including quantum chemical simulations (Paglia et al., 2014; Yesiltepe et al., 2018; Colby et al., 2019), machine learning (Allen et al., 2014; Hufsky et al., 2014; Dührkop et al., 2015; Wolfer et al., 2016; Zhou et al., 2016; Zhou Z. et al., 2017; Zhou Z.W. et al., 2017; Bach et al., 2018), deep learning (Gómez-Bombarelli et al., 2018; Kang and Cho, 2018; Colby et al., 2020), and quantitative structure-activity/property relationship (QSAR/QSPR) models (Wong and Burkowski, 2009; Schneider and Schneider, 2016; Miyao et al., 2017). These approaches dramatically accelerate the library-building process, enabling reference libraries that are orders-of-magnitude larger than those created from analysis of authentic reference material. For example, the IMS-derived molecular property of CCS has been experimentally determined for only 1,884 unique molecules (Colby et al., 2019). *In silico* methods, by comparison, have yielded a predicted CCS library containing over 53 million molecules (Colby et al., 2020).

Compared to experimental reference values, the error inherent in *in silico* predictions does pose limitations to comprehensive, unambiguous identification. However, appropriately modeling this error, as well as the error associated with experimental measurements, enables significant downselection to candidate lists amenable to verification by authentic standards. Further, by leveraging libraries of much broader chemical space coverage, putative matches carry better approximations of false discovery, an until recently ignored metric among the metabolomics community with potentially problematic ramifications (Scheubert et al., 2017; Wang et al., 2018).

Though standards-free approaches to identification in metabolomics studies improve chemical space coverage, and by extension estimates of false discovery rates, libraries are still limited to known chemical space, or, as of this publication, 168 billion molecules, determined from the union of all publicly available databases, including ChEBI, ChEMBL, Enamine, PubChem, UNPD, HMDB, DSSTox, ZINC, KEGG, and GDB17, among others. The “chemical dark matter” that remains is uncharacterizable by techniques discussed thus far. Instead, “library free” approaches, wherein molecular structures can be predicted directly from experimental signatures (i.e., inverse-QSAR/QSPR), must be employed for these “unknown unknowns.” For example, SIRIUS 4 is able to predict chemical structure from mass fragmentation pattern without referencing a database (Dührkop et al., 2019). In addition, nascent advances in generative deep learning approaches have shown promise in Arnold et al. (2016) library free identification (Kadurin et al., 2017a,b; Blaschke et al., 2018; Dai et al., 2018; De Cao and Kipf, 2018; Gómez-Bombarelli et al., 2018; Gupta et al., 2018; Jin et al., 2018; Kang and Cho, 2018; Kim et al., 2018; Lim et al., 2018; Merk et al., 2018; Colby et al., 2020).

In addition, the use of *in silico* metabolism prediction tools will be valuable in expanding the aforementioned databases or chemical search-space to include putative biotransformations of metabolites that result from human or gut microbial metabolism of xenobiotic compounds (Djoumbou-Feunang et al., 2019). Computational strategies that access biosynthetic gene clusters encoded in metagenomic data will be useful for guiding the discovery of novel small molecules from the microbiome (Sugimoto et al., 2019).

## Functional Characterization of the Gut Microbiome Using Activity-Based Protein Profiling

The need to provide direct attribution of microbiota-derived metabolites to specific taxa has brought activity-based protein profiling (ABPP) to the forefront of microbiome science (Whidbey and Wright, 2019; Keller et al., 2020). ABPP exclusively selects for active proteins through function-dependent covalent labeling with small molecule activity-based probes (ABPs), (Cravatt et al., 2008). The ABP-labeled proteins can be subsequently analyzed using mass-spectrometry methods, SDS-PAGE, and live-cell imaging. A new ABP may be hypothetically tailored for any metabolic protein of interest by taking advantage of chemical reactivity or physical binding interactions. The modular nature of an ABP allows for flexibility in post-labeling analysis. ABPP will be an indispensable tool in understanding how microbiome metabolism modulates the host response to external factors, such as diet or environmental exposures.

ABPP acts as a complementary strategy to metaproteomics by circumventing some of the challenges for its application (Heyer et al., 2017; Lee et al., 2017). Metaproteomic profiling provides more functional clues than metagenomics or metatranscriptomics, but the current technologies are not as sensitive as sequencing-based methods. As a result, metaproteomic approaches are biased toward highly abundant proteins, which leaves significant gaps in knowledge. This is particularly relevant to microbiome samples, where certain important taxa are often underrepresented in a population. Sample fractionation and two-dimensional chromatography (capillary and microchip electrophoresis) have been employed to reduce sample complexity (Leary et al., 2013; Tanca et al., 2015; Xiong et al., 2015; Stepanova and Kasicka, 2016). However, this enrichment is costly and thereby limits the number of samples that can be analyzed, and biases the results due to the loss of information (Tanca et al., 2015). However, even with advances in sensitivity, metaproteomic profiles still cannot definitively determine the proportion of the functionally active proteome because many proteins require cofactors, substrates, and post-translational modifications to be functionally active. Because ABPP is inherently an enrichment strategy, it simultaneously retains low abundance proteins and acts as a method to distinguish activity. However, integrating abundance and activity profiles provides a rich representation of the proteome. For example, Wolan and coworkers have shown the potential of coupling ABPP and stable isotope labeling for the enrichment of targeted human and microbial proteins for metaproteomics study in colitis or inflammatory bowel disease mouse model (Mayers et al., 2017).

ABPP also promises to resolve the problem of poorly annotated metagenomes that plagues metaproteomic analyses. It is estimated that 40–70% of the protein coding genes of the human microbiome cannot currently be predicted (Prestat et al., 2014). This problem is exacerbated when the genes come from poorly characterized taxa (uncultured taxa can be up to 40% of metagenomic data). In many cases, genes of unknown function are excluded from analysis because a method that requires mapping to an annotated genome is employed. ABPP can help address this challenge, as ABP-labeling directly signifies protein function (Adam et al., 2004; Kamat et al., 2015; Xu et al., 2015; Martell et al., 2016; Ortega et al., 2018; Elahi et al., 2019). This is especially true when accompanying metabolomics data can provide added confidence (Jansen et al., 2020). ABPP can also identify viable whole cells in complex samples based on a certain function without needing any information beforehand, which may aid in the study of un-culturable microbes with poorly characterized genomes. ABPP has been coupled with fluorescence-assisted cell sorting (FACS) to identify and isolate single cell population responsible for enzyme activity (Whidbey et al., 2019). Hence, combining FACS with ABPP allows for the enrichment of functionally active cells from microbiome with reduced sample complexity. Moreover, these sorted cells can be submitted for further analysis using other omics techniques with a less complex system (Jansson and Baker, 2016).

In addition to the technical advantages of ABPP, the gut microbiome offers many opportunities for exciting conceptual applications because of the diverse chemical transformations mediated exclusively by microbes and their corresponding enzymes and transporters. Host enzymes primarily perform oxidative and conjugative reactions leading to hydrophilic and higher molecular weight metabolites for elimination. In contrast, microbial enzymes typically use reductive and hydrolytic metabolisms to facilitate microbial growth. Many of these reactions can be beneficial for the host, such as the breakdown of plant polysaccharides (indigestible by host enzymes) by complex carbohydrate-active enzymes (>5,000), (El Kaoutari et al., 2013), which can result in the formation of short-chain fatty acid products that can positively influence host health (Rios-Covian et al., 2016). Application of ABPs based on carbohydrates to delineate the role of fiber diet on gut microbiome has a tremendous potential to find robust probiotics in the future (Chauvigne-Hines et al., 2012; Wu et al., 2019).

Function-based profiling will undoubtedly aid in understanding how microbiome activity can bolster host resilience, but it will also be useful for comprehending how it can conversely increase susceptibility to disease. For example, the microbiome produces proteins that degrade the host-produced complex polysaccharide, mucin, whose deregulation is linked to ulcerative colitis (Pullan et al., 1994), and this process has been successfully characterized using ABPP (Tsai et al., 2013; Thuy-Boun and Wolan, 2019). ABPs have also been applied to study the microbiome's modification of other host-produced metabolites, such as bile salts (Zhuang et al., 2017; Parasar et al., 2019), which have implications in the onset of diseases including cholestatic and inflammatory diseases, diabetes, and obesity (Wahlstrom et al., 2016). Microbial enzymes such as proteases, hydrolases, and β-glucuronidases have been labeled using various ABPs and applied successfully in investigating the changes in gut microbiome activity in different disease models (Hatzios et al., 2016; Mayers et al., 2017; Zhuang et al., 2017; Parasar et al., 2019; Whidbey et al., 2019; Jariwala et al., 2020). Importantly, these analyses reveal that change in microbial enzyme activity does not faithfully correspond to gene abundance, which reiterates the necessity of function-based analyses such as ABPP for researchers to harness the chemistry of the microbiome.

## Conclusion

**Figure 1:**
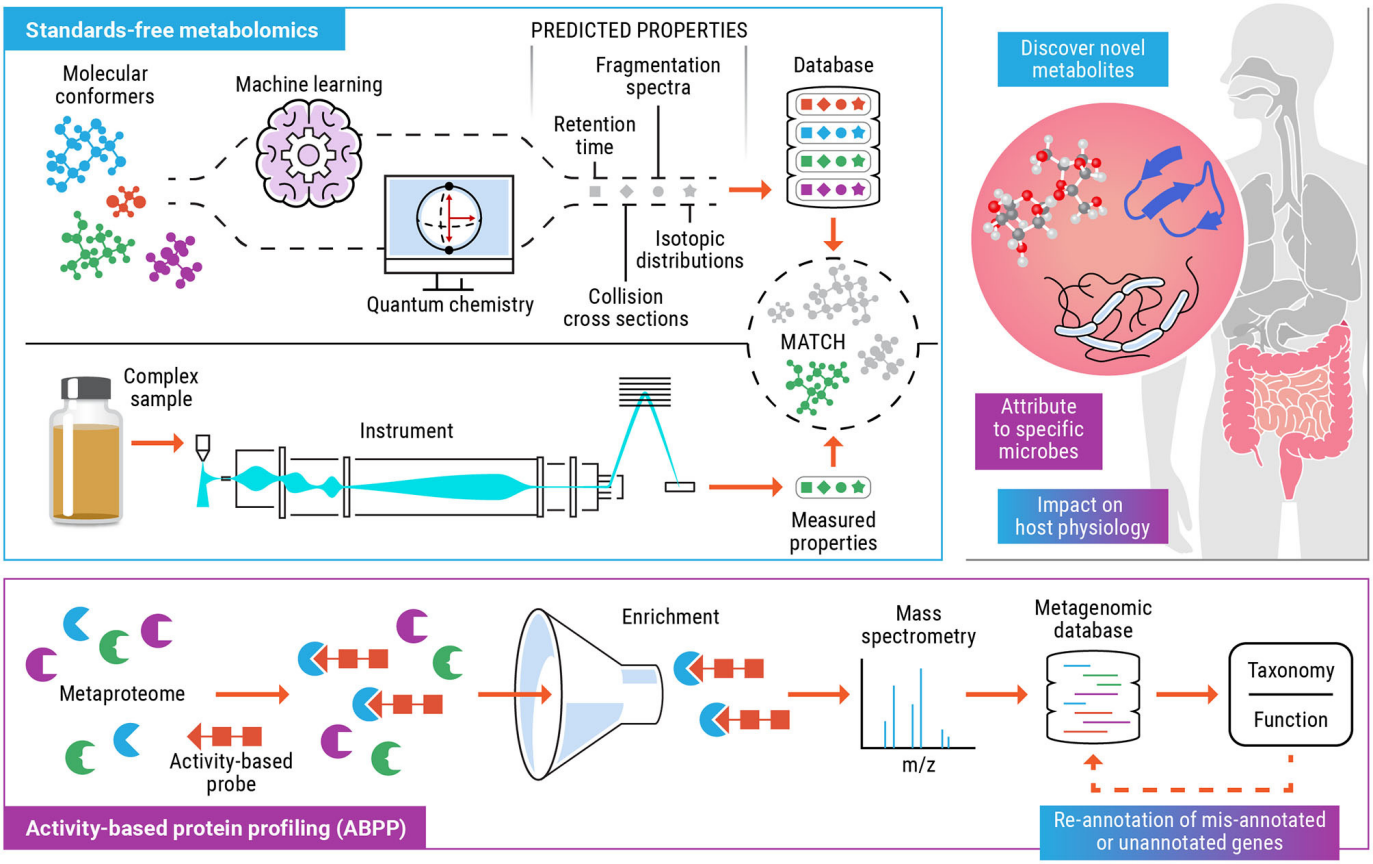
Schematic for the combined use of standards-free metabolomics (blue) and activity-based protein profiling (purple) workflows to functionally characterize biomolecules, the microbial populations involved and their effect on the host. The colored text boxes (blue, purple, or blue+purple) indicate the data and understanding gained from either one or a combination of both technologies.

The objective of this perspective is to highlight the tandem use of standards-free metabolomics and activity-based protein profiling to elucidate the metabolic function of specific taxa and the variety of enzymatic products and small molecule metabolites that they are capable of producing (Figure 1). Comprehensive, untargeted characterization of the metabolome can help identify bioactive metabolites that modulate host phenotype. Activity-based probes can be tailor made for metabolite targets (or dietary compounds or drugs) that have been detected and identified using standards-free metabolomics and implicated to have an impact on the health of the host and the microbiome. This opens up the possibility of categorizing gut microorganisms based on their functional products (enzymes and metabolites), under a defined set of host and environmental factors. We expect that the incorporation of experimental and computationally predicted molecular properties, as part of metabolomics workflows will result in improved detection and increased confidence identification. As researchers start to explore the immense chemical space of human microbiomes and encounter previously unknown molecules, the field will start to rely increasingly on computationally generated libraries containing multiple molecular descriptors such as retention time, CCS, accurate masses of precursor, and fragment ions etc. thus providing increasing confidence of a match as more of these predicted values match with experimentally measured values for a molecule of interest. In recent years, ABPP has emerged as a successful platform to functionally characterize proteins from incompletely annotated genomes and allow study of shifts in functional activity of microbiome in case of change due to external environment, disease, and exposure to chemicals. Standards-free metabolomics coupled with ABPP, ushers in a new era for deciphering the functionally relevant microorganisms in the microbiome. Determining these functional links provides a roadmap for unlocking the full potential of probiotics, developing personalized medicine for individuals based on their unique microbiome, and delineating the relationships between microbial metabolites and human health.

## Author Contributions

SPC and TM conceptualized the work. SPC, NA, SMC, KB, and TM wrote the paper. All authors reviewed and edited the paper.

## Conflict of Interest

The authors declare that the research was conducted in the absence of any commercial or financial relationships that could be construed as a potential conflict of interest.
